# Birth-related long bone fractures in otherwise healthy newborns and medical professional liability: literature review and case presentation

**DOI:** 10.3389/fmed.2025.1589417

**Published:** 2025-08-11

**Authors:** M. Trabucco Aurilio, L. Fava, M. Chisari, M. Bolcato

**Affiliations:** ^1^MESIT Foundation of Social Medicine and Innovation Tecnology, Rome, Italy; ^2^Office of Medical Forensic Coordination, Italian National Social Security Institute (INPS), Rome, Italy; ^3^Rodolico-San Marco Hospital, Catania, Italy; ^4^Department of Medicine, Saint Camillus International University of Health and Medical Sciences, Rome, Italy; ^5^National Blood Centre, Istituto Superiore di Sanità, Rome, Italy

**Keywords:** birth-related fractures, medical professional liability, clinical risk, long bone fracture, malpractice

## Abstract

Birth-related long bone fractures are rare but clinically significant events that require careful evaluation to distinguish them from fractures caused by underlying pathological conditions or non-accidental trauma. Their diagnosis and management have important clinical and medico-legal implications. A selective literature review was conducted to identify relevant studies published between 2004 and 2024, regarding incidence, mode of delivery, fracture location, time to diagnosis, treatment, and outcomes. Additionally, an original case report of a female neonate diagnosed with femoral shaft fracture on the third day of life. Neonatal long bone fractures can occur even in the absence of predisposing genetic or metabolic conditions. While they generally heal without for surgical intervention, timely diagnosis through appropriate imaging is crucial to ensure proper management. Failure to do so may expose healthcare professionals and institutions to potential medico-legal liability, both during delivery and in the immediate postnatal period. Preventive strategies should focus on careful prenatal risk assessment, adherence to best practices in delivery maneuvers, and early postnatal monitoring to optimize outcomes and minimize legal risks.

## 1 Introduction

Birth-related fractures are an infrequent but significant complication of delivery, with an estimated incidence of approximately 2.9 per 1,000 live births, according to the latest data (1, 2). Among these, clavicular fractures are the most commonly reported, and frequently associated with challenging deliveries, such as those involving shoulder dystocia, breech presentations, or excessive traction during birth. These fractures are often accompanied by brachial plexus injuries, further complicating neonatal outcomes (3). While fractures of the skull, ribs, and long bones are substantially rarer, they remain a recognized possibility, particularly in traumatic or instrumental deliveries (4).

Neonatal long bone fractures typically involve the femur, humerus, or tibia and predominantly occur during vaginal deliveries. However, several cases of long bone fractures have been documented in the context of cesarean deliveries as well (5, 6). These injuries are more frequently observed in neonates with severe congenital or malformations, such as myofibromatosis, osteogenesis imperfecta, arthrogryposis, and muscular dystrophies. Such underlying conditions predispose newborns to fractures even under minimal stress during delivery (7–9).

However, although rare, cases of long bone fractures have been reported in otherwise healthy newborns without malformative conditions (4). In such instances, professional liability may arise for healthcare personnel involved in delivery or in subsequent postnatal management.

The purpose of this study is to review the literature on birth-related long bone fractures in neonates without malformation or genetic or dysmetabolic conditions, present a clinical case, and discuss the medico-legal implications concerning medical professional liability.

## 2 Case presentation

We report the case of a female neonate born at 35 weeks and 4 days of gestation due to preeclampsia and a previous cesarean section, which led to the decision to perform an urgent repeat cesarean delivery. The fetus was diagnosed with intrauterine growth restriction (IUGR), with fetal doppler showing the umbilical artery flow at the 95th percentile and the cerebro-placental ratio at the 5th percentile, accompanied by abnormal cardiotocographic findings. The fetus was delivered in a cephalic presentation, and no noticeable complications were encountered during the procedure.

After birth, the neonate was entrusted to the pediatric department for initial care and cleaning, followed by placement in an incubator. On the third day of life, the neonate was found to have significant edema in the right lower limb, which was positioned in flexion and abduction, with no active movement of the thigh on the pelvis. Upon palpation, a hard swelling with crepitus was noted in the middle third of the thigh.

Further investigation through radiography revealed a fracture of the right femoral diaphysis. An orthopedic consultation was performed, which documented swelling of the thigh, absent spontaneous mobility, and movement of the foot upon stimulation and mobilization. The leg was placed in traction with a cast, which was maintained for approximately 15 days. The neonate underwent several orthopedic follow-ups, and after 2 months, no significant permanent sequelae were observed.

## 3 Methods

A selective literature review was conducted using Medline (PubMed) to identify relevant articles published in the last 20 years.

The search was performed with the following keywords: “newborn long bone fractures,” “neonatal long bone fractures,” and “birth-related long bone fractures.” Only articles published in English between 01.01.2004 and 31.12.2024 were included. Publications predating 2004 were excluded, along with studies related to fractures caused by underlying genetic or malformative conditions (e.g., osteogenesis imperfecta, myofibromatosis, muscular dystrophies) and articles focused on fractures related to child abuse. We also excluded articles for which the full text was not available.

Two of the reviewers carried out the initial search of the papers. They used the search protocol described above to identify literature. In the case of disagreements, the consensus of the research supervisors was asked. The researchers used the following research order: titles were screened first and then abstracts and full papers. A paper was considered potentially relevant and its full text reviewed if, following discussion between the two independent reviewers, it could not be unequivocally excluded on the basis of its title and abstract. The full text of all papers not excluded on the basis of abstract or title was evaluated.

A database was created from the selected studies, focusing on the following parameters: article type, the number of cases (excluding those related to congenital, genetic or metabolic comorbidities or abuse), mode of delivery (cesarean, vaginal or not specified), fracture location, time interval between birth and diagnosis of the fracture, treatment methods, and any permanent sequelae.

## 4 Results

The literature research resulted in a total of 22 suitable papers, including 5 retrospective cohort studies and one population-based study. Key findings are summarized in Table 1.

**Table 1:** Summary of literature review findings.

Article	Type	Number of cases*	Delivery (n)	Fractures location (n)	Birth-diagnosis time span	Treatment	Permanent sequelae
Basha et al. (4)	Retrospective	6	Vaginal (1), Cesarean (7)	Femur (4) Humerus (2)	1–3 days	Immobilization (soft splintage)	None
Canpolat et al. (6)	Case report	1	Cesarean	Humerus bilateral	6 h	Immobilization	None
Capobianco et al. (18)	Case report	1	Cesarean	Femur	1 day	immobilization	None
Cebesoy et al. (19)	Case report	1	Cesarean	Femur (bilateral)	2 days	Immobilization (pelvipedal cast)	None
Dias (20)	Case report	1	Cesarean	Humerus bilateral	Immediately	Immobilization (splintage)	None
Farikou et al. (21)	Case report	1	Cesarean	Femur	Immediately	Immobilization (bilateral contention)	None
Galeotti et al. (2023) (13)	Case report	10	Vaginal (9), Cesarean (1)	Humerus (10)	0–9 days	Close reduction and cast immobilization; close reduction and percutaneous pinning with Kishner wires (2 cases)	Occasional elbow pain during sports (1 case) Transient fifth finger paresthesia (1 case)
Givon et al. (22)	Case report	9	Vaginal (1), Cesarean (8)	Femur (7) Femur bilateral (2)	Unknown	Immobilization (Bryant splint)	None
Goyal et al. (23)	Case report	1	Cesarean	Humerus	2 days	Immobilization	None
Hamilçıkan et al. (24)	Case report	1	Vaginal	Humerus	Immediately	Immobilization	None
Hosokawa et al. (25)	Retrospective	7	Not specified	Femur (7)	Unknown	Unknown	Unknown
Kanai et al. (5)	Case report	1	Cesarean	Femur	9 days	Immobilization cast	Unknwon
Kanat Pektas et al. (26)	Retrospective	5	Cesarean	Femur (5)	1 day	Immobilization	Unknown
Kaya et al. (27)	Case report	1	Vaginal	Humerus	Immediately	Immobilization	None
Kim et al. (28)	Case report	1	Cesarean	Femur	Immediately	Immobilization (splint)	None
Matsubara et al. (29)	Case report	1	Cesarean	Femur	Immediately	Immobilization (cast)	None
Mileto et al. (10)	Case report	1	Cesarean	Tibia	Immediately	Percutaneous pinning K wires	None
Rahul et al. (2017) (16)	Case report	1	Cesarean	Femur and humerus	2 days	Immobilization	None
Rehm et al. (11)	Retrospective	15	Not specified	Humerus (13) Femur (1) Tibia (1)	Unknown	Immobilization K wires pinning (1)	Unknown
Rehmani et al. (30)	Retrospective	3	Not specified	Femur (1) Humerus (2)	Unknown	Immobilization	None
Toker et al. (31)	Case report	1	Cesarean	Femur	10 days	Immobilization	None
Von Heideken et al. (12)	Population-based	210	Not specified	Femur (30) Humerus (180)	1–7 days	Unknown	Unknown

* Excluding those associated with congenital, genetic or metabolic comorbidities or abuse.

The analysis of the selected studies identified a total of 279 cases of neonatal long bone fractures. These cases were distributed as follows: 210 cases of humeral fractures (75.3%), 61 cases of femoral fractures (21.9%), 2 cases of bilateral femoral fractures (0.7%), 2 cases of bilateral humeral fractures (0.7%), 2 cases of tibial fractures (0.7%), and 1 case involving simultaneous fractures of the femur and humerus (0.4%).

In most cases (*n* = 243), the mode of delivery was not reported. Among the cases with available data, 13 fractures occurred following vaginal deliveries, while the remaining cases (*n* = 58) were associated with cesarean sections. The time interval between delivery and diagnosis of the fractures ranged from immediately after delivery to 10 days postpartum.

Practically all fractures were treated conservatively, using methods such as immobilization or splinting. Only 3 cases (1.1%) required surgical intervention, specifically percutaneous pinning with Kirschner wires. Most fractures healed without permanent complications. However, two cases reported minor sequelae: one patient with a humeral fracture experienced occasional shoulder pain during sports activities, and another patient with a humeral fracture reported transient paresthesia in the fifth digit of the hand.

## 5 Discussion

The occurrence of birth-related long bone fractures, although rare, represents a clinically significant complication with implications for neonatal outcomes and potential medico-legal consequences, especially in newborns with no associated comorbidities. A few studies have highlighted the critical need to distinguish these fractures from those associated with child abuse (2). Furthermore, these cases carry notable medico-legal implications in the context of medical professional liability.

Our review of the literature revealed that both vaginal birth and cesarean section could be responsible for birth trauma, including long bone fractures.

Among the 279 cases analyzed, humeral fractures were the most frequent, followed by femoral fractures, with bilateral fractures and tibial fractures being exceedingly rare (10, 11).

The predominance of humeral fractures aligns with their anatomical vulnerability during delivery, particularly in challenging vaginal deliveries (6, 12). Interestingly, a notable proportion of fractures occurred during cesarean sections, highlighting mechanical forces applied during extraction can still result in neonatal skeletal injuries, even in controlled surgical settings. This underscores the need for heightened vigilance and careful manipulation during delivery.

One of the most intriguing findings relates to the highly variable time span between delivery and the diagnosis of neonatal fractures. While most cases were identified immediately after birth or within the first 2–3 days postpartum, some diagnoses were delayed for up to 10 days (5, 13). It is well-established that diagnosing fractures in neonates can be particularly challenging, as these injuries often manifest with nonspecific symptoms such as irritability, excessive crying, or feeding difficulties. Such delays in diagnosis, or the premature discharge of neonates without identifying the fracture, can raise significant medico-legal concerns. Furthermore, while fractures diagnosed immediately after birth can be directly linked to maneuvers performed during delivery, the determination of when the injury occurred becomes more complex when the diagnosis is made from the second or third day postpartum onwards.

Due to the nonspecific nature of clinical presentations, it can be difficult to discern whether the fracture was sustained during delivery but went unrecognized or occurred later as a result of improper handling, suboptimal care, or trauma during hospitalization. This ambiguity complicates the attribution of responsibility among the healthcare providers involved, posing substantial challenges in medico-legal assessments.

The clinical case presented exemplifies the challenges highlighted. Clinical records analysis did not identify any evident complications during the cesarean delivery. The diagnosis of the fracture was made 3 days after birth, triggered by the onset of significant swelling in the lower limb. From a medico-legal standpoint, this case underscores the difficulty in pinpointing the timing and mechanism of the fracture. It remains uncertain whether the injury occurred during delivery or in the days that followed, and the precise trauma responsible cannot be definitively determined. As a result, establishing liability for the healthcare professionals involved is particularly challenging in such circumstances.

One way to improve diagnostic accuracy in neonatal fractures may be to apply novel imaging techniques. Traditionally, X-ray has been the standard due to its low cost, speed, and availability. However, it has several drawbacks, including the need to reposition the infant—potentially worsening the injury—longer exam times, radiation exposure to both patient and staff, and a risk of missed diagnoses. Ultrasound, though not routinely used in this context, has recently emerged as a promising alternative. It is accurate, non-invasive, radiation-free, and allows for bedside assessment without moving the infant, thus reducing the risk of aggravating the fracture. Studies have shown its efficacy in detecting rare injuries such as Salter-Harris type I fractures of the distal humerus (14). Moreover, a recent meta-analysis highlighted its high reliability in diagnosing neonatal clavicle fractures, suggesting that ultrasound could be considered a new gold standard in selected cases (15).

Finally, findings from our review demonstrate that the vast majority of neonatal long bone fractures heal without permanent sequelae when managed conservatively, as in the case presented. This aligns with the literature emphasizing that prompt and appropriate treatment, such as immobilization or traction, is typically sufficient to achieve favorable outcomes. Surgical intervention was required in a residual number of cases, reflecting its limited necessity in this population (13, 16). From a medico-legal perspective, even if negligence on the part of healthcare professionals were to be established, the resulting claims for personal injury would generally be limited to minor temporary consequences. In only the rarest cases would there be minimal, permanent functional impairments, like transient paresthesia or Occasional pain during sports activities (13).

## 6 Conclusion

Neonatal long bone fractures are uncommon but can occur even in the absence of predisposing genetic or metabolic conditions. While most cases do not require surgical intervention and heal without permanent sequelae, accurate radiological assessment is essential to ensure prompt and appropriate management. Failure to establish a timely diagnosis and initiate proper treatment may expose healthcare providers and institutions to potential medico-legal liability, both in relation to the delivery process and the immediate postnatal care of the newborn.

The results of this review show how in many cases there is a significant diagnostic delay and therefore greater clinical and medical-legal risk (17), for this reason the knowledge of this study can allow greater awareness of the operators and timely intercept a possible fracture avoiding further damage and, in many cases, prevent them.

## Data Availability

The datasets presented in this article are not readily available. Requests to access the datasets should be directed to fava.ludovico@gmail.com.
